# Dynamic Optimization Modeling of Smart Tourism Information System Using VRGIS in Big Data Environment

**DOI:** 10.1155/2022/7914674

**Published:** 2022-10-10

**Authors:** Anfeng Xu, Wenjun Zeng

**Affiliations:** ^1^Economics and Management School of Harbin University of Science and Technology, Harbin 150080, China; ^2^Key Laboratory of Island Tourism Resource Data Mining and Monitoring, Ministry of Culture and Tourism, Sanya 572099, China

## Abstract

The establishment of an intelligent, comprehensive, and all-encompassing information system for tourism management is the current trend in tourism informatization as a result of the continual development of modern information technology. Significant advancements in the field of VRGIS and its usage in research have been made as a result of the use of VRGIS to categorize, assess, plan, and manage tourism resources. The analysis of the recent development of VRGIS in tourism resource research is the first section of this work. This study examines and implements a mobile, computerized, and intelligent tourism service system that gives visitors a sense of the surrounding landscape using VRGIS. Three-dimensional mapping, environment detection, personal trajectory, and Weibo sharing are just a few of the system's many helpful features. While travelling, tourists can get services that are more intelligent and practical. The drawbacks of conventional geographic information systems include their reliance on sophisticated models, network limitations, and operational challenges. New software architecture is put in place to get rid of network restrictions, virtual reality peripherals are used to make operation more convenient, and system modeling is rebuilt using the TIN data model and model simplification. The results of experiments show improved refresh rates and peripheral expansion modules. The user experience is enhanced by this research.

## 1. Introduction

The tourism industry has become an essential driver of growth in a great number of nations, and the tourism resources that are available are an essential component of the material foundation on which the tourism sector is built and maintained [1, 2]. In order to provide a fundamental basis for the evaluation, planning, and reasonable protection of tourism resources, it is essential to recognize, classify, collect, and build a database of tourism resources. This is carried out in order to provide a fundamental basis. As a result of the one-of-a-kind qualities of tourism resources, the theory and methods of VRGIS are one-of-a-kind when it comes to spatial cognition and the design of classification systems, as well as the standardization of databases, as well as spatial analysis and evaluation analysis [3–5]. It is now an accepted method and an integral part of the entire process of researching, developing, and making use of tourism resources that this practice has become a common place when carrying out research on tourism resources. The utilization of VRGIS in tourism research and application has important enlightenment significance for both the understanding of the theoretical and technical system and the guiding of the utilization of tourism resources[6].

Since the beginning of time, one of the most vital areas of research has been focused on the application of GIS in the process of developing and managing tourism resources. The study of tourism resources has made extensive use of GIS. In recent years, the tourism industry has begun to make use of geographic information systems, more commonly referred to as GIS. There is a strong connection between the theory and technology of GIS and the investigation and utilization of tourism resources[7–9]. The vast majority of researchers working in this area have concentrated their attention on the function of GIS in the cultivation and application of tourism resources; however, a few of them have also carried out pertinent review research in this sector. There is still a lack of systematic application of GIS in the development and management of tourism resources, which leads to issues such as a lack of application and a lack of understanding of application fields. One solution to this problem is to increase the use of GIS in tourism resource development and management[10–12].

Due to the rapid advancement of information technology in the modern world, the current trend toward the informatization of tourism involves the extensive application of cutting-edge technologies such as VRGIS. Due to advancements in mobile Internet technology, high-speed networks are becoming more portable, which contributes to their growing popularity. The integration of smartphones with information search, communication, entertainment, and social networking provides users with access to dynamic information services and personalized recommendation services [13–17]. The traditional tourism information management system has a number of issues, such as a lack of usability flexibility, a one-dimensional information display, and a subpar user experience maintaining our forward momentum. Rapid advancements in virtual reality (VR) and augmented reality (AR) technologies in recent years have made it possible to show user-required information in multiple temporal and spatial dimensions, as well as to perform highly immersive interactive activities. New concepts for the future growth of the tourism industry have been inspired by these advances. VR offers customers a realistic and engaging visual experience through its visual sense [18, 19]. Virtual and physical aspects are combined in augmented reality (AR), which is based on VR. By including text, pictures, voiceover, and any other relevant information, you may help the user become more aware of and understand the real world. A new technology based on mobile platforms is starting to emerge as smart device performance keeps getting better: VRGIS technology. Additionally, mobile VRGIS is less confined by the space environment and is more versatile and portable than standard virtual-real integration. The VRGIS technology that is based on mobile platforms has been successfully used in a wide range of fields (including security, education, and urban management) [20, 21], and as a result, it provides an invaluable resource for resolving the issues that the tourism information service industry is currently experiencing. To accomplish the goals of a mobile VRGIS tourist system, mobile intelligent terminals must incorporate VRGIS technology [4, 22–24].

Traditional tourism is based on a two-dimensional map, but this type of vacation offers little in the way of experience or interaction, and there is room for improvement in the areas of information acquisition and spatial analysis. There are still some travel-related services that can only be accessed online, specifically via websites. Due to a lack of adaptability, real-time travel services are currently unavailable. Due to the fact that many modern systems only provide a single navigation or scene-reproduction function, they cannot be user-friendly or interactive. This is because they only perform a single function. This study creates a VRGIS-based tourism system for mobile platforms with the intention of enhancing the system's usability and interactivity.

## 2. Background

The utilization of big data has made possible the opening of new lines of inquiry into the collection and analysis of data pertaining to conventional tourist destinations. Creating a tourism resource directory that is based on big data and developing a semantic rule extraction model for key attributes can significantly cut down on the amount of time spent out in the field. The tourism system performs a combined evaluation based on a particular weighting by utilizing a big data semantic analysis and statistical analysis, in addition to a big data expert scoring mechanism. Data from social media platforms that pertain to the travel of tourists, such as the latitude and longitude of each point of interest (POI), the content and travel notes of each POI, the number of travel comments received by each POI, and its score, are used to take part in the evaluation of tourism resources and to improve the scientific nature of public participation. Some researchers believe that the train collection head can be used to collect data about the popularity of tourist destinations within the comment network [7, 11]. This data can then be used to study the relationship between search popularity and comment popularity. The Internet is utilized in order to collect large amounts of information from a selected group of scholars, such as the number of comments and the overall rating of a tourist city, while analysis is utilized in order to determine the individual ratings of various types of tourism resources. After that, GIS software can be used to measure and assess the tourism industry's resources. Some researchers concentrate their attention on rural tourism resources and make use of geospatial big data in order to conduct a spatial classification of rural tourism within tourism development zones [4, 15, 17, 19, 21].

To account for the influence and contribution of these factors, it is necessary to consider the overall potential assessment of regional tourism resources, as well as spatial characteristics such as scale, scope, structure, spatial correlation, and pattern pedigree. It is necessary, when evaluating tourism resources, to improve comparability between different evaluation methods and to compare and optimize various algorithms and results. In addition, it is necessary to enhance the comparability of various evaluation methods.

It is important to evaluate the aggregation, structure, pattern, and distribution law of tourism resources in space when examining regional tourism resources. This includes factors such as the quantity and density, type and quality structure, and combination and spatial correlation of tourism resources within a specific spatial range, as well as other factors of a similar nature. Construction of a prototype from scratch: more than a dozen distinct GIS methods have been used to evaluate a region's tourism resources. Models for evaluating the availability of resources in gathering areas, agglomeration areas, and combination areas are included in these methods. These models consist of buffer and proximity analysis, network and overlay analysis, and combination area analysis, among others. In tourism planning, some academics have utilized the GIS multiscale grid modeling technology to extract the spatial map of tourism resources, as well as significant points, lines, and areas of high-quality tourism resource areas. This has strengthened scientific planning.

The administrative unit scale and the tourist area scale are the two primary scales that are utilized when evaluating the tourism resources of a regional area. When carrying out spatial modeling, they only use statistical indicators as their basis for evaluating the tourism resources offered by the region. An examination of the manner in which tourism resources are dispersed geographically, as well as the spatial correlation between the resources' quality, will be carried out, along with an investigation into the methods for extracting the spatial map of the region's tourism resources and designing tourism resources for tourism products. There has not yet been any work carried out to create and implement potential value knowledge maps. To make matters even more confusing, no attempt has been made to take into account nonadministrative geographic areas, such as coastal zones or geomorphic units (such as mountains and valleys), in order to include tourism resources. This makes the situation even more difficult. If any consideration of the spatial relationship between tourism resources is given at all, it is in the form of a qualitative description. This is in stark contrast to the extremely limited attention that is paid to the issue. Combining traditional mathematical models with GIS data is the primary foundation upon which the evaluation method rests.

## 3. System Design

The system design is as follows.

### 3.1. Architecture Design

**Figure 1:**
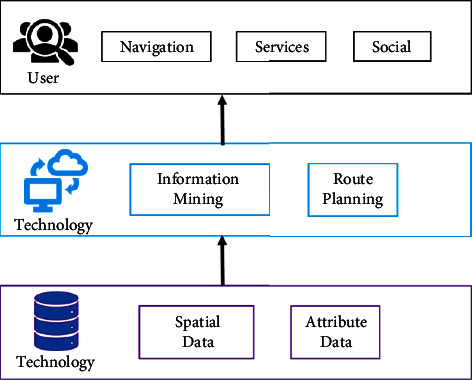
Structure of intelligent tourism management system.

The system implements route planning and virtual roaming using three-dimensional landscape maps and geospatial data from tourist attractions. Users can do this to get a firsthand view of the scene and learn more about the route and beautiful sights before they journey there. The virtual environment, images, and text labels are flawlessly integrated into the real world seen by tourists through the use of location-based service technology and augmented reality technology. This is carried out to present visitors with a fresh viewpoint on cognition and an interactive application experience, two things that conventional software cannot offer.  Figure 1 illustrates the system's architecture.

The user layer is the topmost layer. Some of the system's most crucial operations, such as virtual roaming and route planning, are made possible by the usage of a three-dimensional landscape map as a map base. The system is responsible for doing these tasks. Thanks to AR technology, the intelligent service module can recognize its surroundings and show a 3D model of an object. At the same time, a heat map depicting the movement of individuals through the picturesque region is created utilizing GPS positioning and data mining techniques. Users can share social media items such as their own footprints, those of their friends, and connections to other apps such as Weibo through the social module, for the most part. The footprints of friends are another illustration of this kind of stuff.

Technical elements make up the second layer of this structure. In essence, the system's technology consists of two distinct components. With the use of location, multisensor tracking, and registration technology, spatial data may be obtained and analyzed. Opportunities for spatial analysis and spatial data mining arise as a result. GIS spatial analysis and data scheduling are used to execute three-dimensional virtual navigation and route planning on the map, which is based on a three-dimensional landscape map.

The data layer is the topmost layer. The system data centre is made up of two distinct parts: a geospatial database and a GIS spatial database of picturesque sites. The road network, trajectory, and position of scenic locations are all included in the GIS spatial database for scenic spots. One feature that can be used in data is text; additional examples include photographs and videos.

### 3.2. Geographic Information Modeling

**Figure 2:**
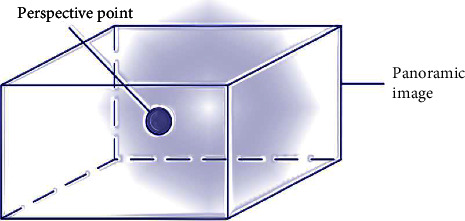
Panoramic image observation model.

This study makes use of it, and a panoramic camera is used to capture all of the images in an area as textures so that fitting processing can be carried out later. The main perspective of the observer is encompassed by the panoramic image, which enables the observer to experience what it is like to be immersed in the area and enhances the feeling of being immersed. A representation of the panoramic image observation model can be seen in Figure 2.

**Figure 3:**
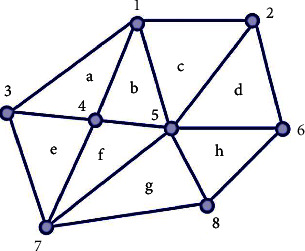
Topology diagram.

This research makes use of a digital elevation model, also known as a DEM, to model the uneven surface and the buildings because it is impossible to describe the area using a straightforward two-dimensional plan. In order to accomplish the goals of this investigation, a model consisting of irregular triangles was utilized. Because each point in the TIN model needs to be stored with its elevation coordinates and topology, the TIN model is more realistic than its DEM counterpart, which is one reason why it is more reliable in data storage. The representation of a region's topology as it appears in the TIN model is shown in Figure 3. This representation keeps track of every triangle, face, and point in the area.

An extension of the logistic regression classification known as the Softmax regression classification method is utilized in this study in order to better solve the multiclassification problem. This is due to the fact that the classification of human actions is a multiclassification that is mutually exclusive.

The area in Figure 3 is marked as follows:

(1) $$\begin{array}{c} ar=\left\{a,b,c,d,e,f,g,h\right\}\text{.} \end{array}$$

Vertices in the graph are marked as follows:

(2) $$\begin{array}{c} ve=\left\{1,2,3,4,5,6,7,8\right\}\text{.} \end{array}$$

Then, we obtain the triangle file as shown in Table 1.

By eliminating insignificant points, lines, and surfaces from the model's geometric pattern, it is possible to cut down on the number of faces the model has. By utilizing the vertex deletion method and the progressive mesh simplification method, this can reduce the amount of running data required by the model, while simultaneously increasing the refresh rate. When deciding whether or not to remove the inner central vertex, one of the most important factors to take into account is how this will affect the image's position in the plane. Here is the formula you need to use to calculate the results:

(3) $$\begin{array}{c} F=\left|k\left({Point}_{i}-C\right)\right|\text{,}\\ k=\frac{K}{\left|K\right|}\text{,}\\ K=\left({Point}_{i}-{Point}_{i+1}\right)\left({Point}_{i}-{Point}_{j}\right)\text{.} \end{array}$$

The calculation method of C is related to the simplified triangle *a*_*i*_:

(4) $$\begin{array}{c} C=\frac{\sum x_{i}a_{i}}{\sum a_{i}}\text{,}\\ x_{i}=\frac{{Point}_{5}+{Point}_{1}}{2}\text{,}\\ a_{i}=\Delta {Point}_{5}{Point}_{1}\text{.} \end{array}$$

The sensor rotation matrix is as follows:

(5) $$\begin{array}{c} \left[\begin{array}{ccc} S\left(0\right) & S\left(1\right) & S\left(2\right)\\ S\left(3\right) & S\left(4\right) & S\left(5\right)\\ S\left(6\right) & S\left(7\right) & S\left(8\right) \end{array}\right]\text{.} \end{array}$$

The system makes use of tile map technology and displays the three-dimensional map of the landscape in Mercator projection. This is carried out so that the map can be seen in its full three-dimensional glory. First, a model of the Wutai Mountain scenic area is constructed with the help of virtual reality in order to generate an accurate landscape map. After that, you must tile the images after cutting the landscape map into squares, comparing and correcting them with the overall map that you downloaded from Baidu, and then finally tiling them. Second, the indexed and published cut map tiles are arranged using the WMS interface standard. The server side will return the tile data that corresponds to the received data range once it has parsed the data that was transmitted by the mobile terminal. The map can now be completely scheduled by the mobile terminal as a consequence of the real-time splicing and rendering that has taken place. The coordinates are then transformed, as the last step. In order to ensure that the actual latitude and longitude are used for navigation, a formula needs to be used to perform a transformation on them before they can be projected onto a map of a three-dimensional landscape.

Using VRGIS spatial analysis and the Freud shortest path algorithm, this system implements the navigation function. In addition to the conventional two-dimensional vector map navigation, it provides users with a mode for virtual roaming. In addition, the system dynamically adjusts the rotation angle of the map so that it corresponds with the user's current direction and range on the mobile terminal screen beneath the top view of the three-dimensional landscape map, which is monitored in real time by the mobile sensor. In the context of the three-dimensional landscape map, this is visible beneath the map's top view.

This system employs the outdoor 3D registration technology of mobile terminals in order to realize the authenticity and real-time capabilities of the mobile virtual reality system. This technology is based on the mobile augmented reality module's positioning recognition. This technology enhances the real-time capabilities, stability, and robustness of 3D environment registration, along with the authenticity and fusion of the virtual-real combination scene. It focuses primarily on the spatial positioning of the camera and the placement of the virtual object in actual space. Based on these details, the rendering of the virtual object is completed.

## 4. Results

Utilizing a distributed system architecture, the system deploys gateway services, map data services, and scenic spot information data services on multiple Internet cloud servers. As a direct result, the system will be able to accommodate the exponential increase in user numbers. Distributed system operation is greatly aided by cohesion and transparency that are at a high level. The network can be used to establish a distributed cluster framework that grants each service node a high level of autonomy. The cluster framework has fewer moving parts, which makes it easier to maintain and simpler to operate. As a direct result, from the user's perspective, the distribution of each node to the user's application is completely obscured. The distributed network architecture eliminates the need for users to be concerned with complex relationship structures and synchronization requirements when accessing data that is distributed across multiple locations. Using the gateway service, a master control server will distribute a large number of service requests to individual servers when it receives a large number of service requests. This will decrease the amount of computational stress the system experiences.

**Figure 4:**
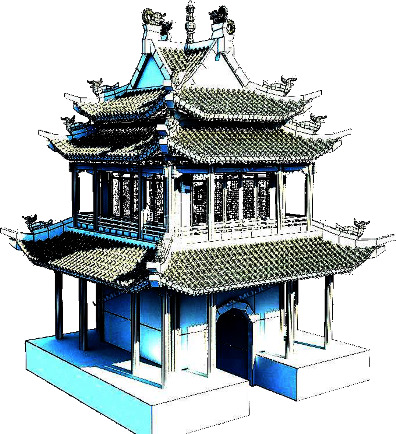
3D map display.

**Figure 5:**
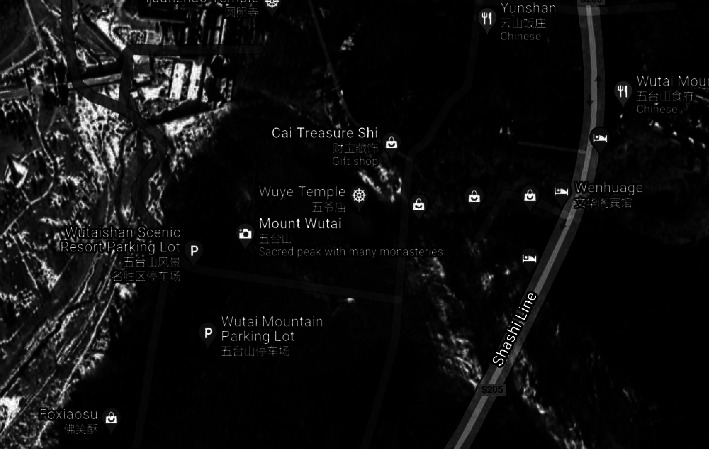
Route navigation map.

This mode displays a three-dimensional landscape map of the entire Wutai Mountain scenic area. Users may zoom in, move around, and access more information in this mode. The landscape map may be rotated while you are moving around, and the labels for each picturesque spot will do the same. The user's perspective remains unchanged from the frontal angle when they enter roaming mode. The designation of the beautiful area serves as the starting point for route planning, as shown in Figures 4 and 5. Additionally, it is possible to plan routes to a variety of beautiful places, and if desired, the tour route can be researched beforehand. In particular, the system we have created has descriptions of attractions in both Chinese and English.

In the environment recognition module, users can click on a specific scenic spot on the map, and the system will automatically pop up the specific picture and introduction in Chinese and English of the scenic spot. Users can also upload photos of the place, as well as make reviews and ratings.

**Figure 6:**
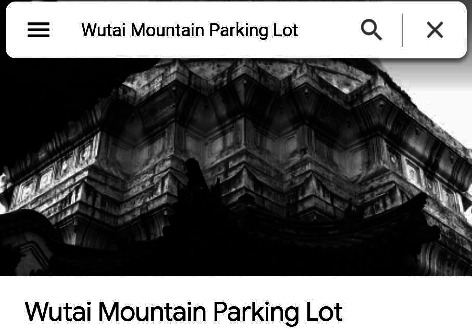
Specific classic search module.

In order to produce information-rich queries, prosperous scenic spots, key monitoring areas, and important landmark buildings in the vicinity of the scenic spots are used to conduct specific queries. These queries are based on the scenic spots that are important to the user's interests and are used to conduct specific queries. Through the standardized processing of queries such as landmark queries, business district queries, catering queries, entertainment place queries, traffic route queries, fire protection facility attribute queries, water conservation facility attribute queries, and business data queries, it is possible to integrate the business management of 3D visualizations of scenic spots into 3D cities, as shown in Figure 6.

**Figure 7:**
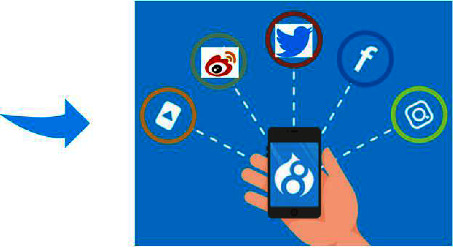
Share module.

In the social module, users can share their route maps, tourist photos, etc. The tourism system also supports other contact APP logins, saving users the trouble of registering separately, as shown in Figure 7.

## 5. Discussion

Participation by the tourism industry and the general public in VRGIS projects, as well as the number of tourism systems employing this technology, are on the rise. According to the findings of previous studies, academics who work in the field of tourism and geographic information systems (GISs) appear to be the primary sources of information used to analyze and evaluate tourism resources. The latter emphasizes the evaluation's content in addition to its outcomes. When evaluating or analyzing tourism resources, the analytic hierarchy process (AHP) places a greater emphasis on spatial thinking patterns and technological innovations than the national standard method, which is the more prevalent technique. In recent years, an increasing number of VRGIS researchers have focused their efforts on tourism resource-related research. This type of research examines census technology for tourism resources, tourism resource analysis and evaluation methods, tourism resource development planning, and technology for analyzing big data in tourism.

### 5.1. Developing and Perfecting the Research Methodology

As the growth of tourism resources' transitions from a key development stage to a comprehensive development stage, the rise of global tourism, and the iterative update of intelligent and portable mobile terminal equipment, an increasing number of industries, businesses, and members of the general public will become involved in the recognition of tourism resources.

The method system can be innovatively enhanced with the aid of VRGIS and a tourism knowledge model. Future tourism resource big data research hotspots will include scientific tourism resource spatial cognitive models, tourism resource graph knowledge models, tourism resource development case models, and tourist destination selection behavior models. There will be a new and improved method for evaluating the available tourism resources, which will be based on a set of semantic rules derived from spatial thinking, as well as relevant maps.

In Section 1 of this study, we will examine the rapid growth of VRGIS in tourism resource research over the past several years. Mount Wutai, a Buddhist holy site, is used as an example to illustrate how VRGIS can be used to develop a mobile, informaticist, intelligent, and personalized tourism service system that provides tourists with a better understanding of the surrounding landscape.

### 5.2. An Original Perspective and Method of Interacting with the Outside World

The system consists of a three-dimensional landscape map, recognition of the surrounding environment, tracking of a user's trajectory, and the capacity to share information via Weibo. On the road, tourists now have access to services that are both more knowledgeable and more convenient. Traditional geographic information systems have several drawbacks, including operational challenges that are difficult to use, complex model structures, and limited network capacities. System modeling is reconstructed with the aid of the TIN data model and model simplification; a new software architecture is implemented to eliminate network constraints; virtual reality peripherals are utilized to make operation more straightforward and convenient. Experiments conducted for this study demonstrate that this work enhances the user experience by increasing the number of peripheral expansion modules and the system's refresh rate.

## Figures and Tables

**Table 1:** Detail of triangle file.

Area point	Vertex			Collar triangle		
A	1	3	4	—	e	B
B	1	4	5	a	f	C
C	1	2	5	—	d	B
D	2	5	6	c	h	—
E	3	4	7	a	f	—
F	4	5	7	b	g	E
G	5	7	8	d	—	H
H	5	6	8	d	—	G

## Data Availability

The data used to support the findings of this study can be obtained from the corresponding author upon request.
